# Contributions of myofascial pain in diagnosis and treatment of shoulder pain. A randomized control trial

**DOI:** 10.1186/1471-2474-10-92

**Published:** 2009-07-24

**Authors:** Sara Perez-Palomares, Bárbara Oliván-Blázquez, Ana Mª Arnal-Burró, Orlando Mayoral-Del Moral, Elena Gaspar-Calvo, Mª Luisa de-la-Torre-Beldarraín, Elena López-Lapeña, Marina Pérez-Benito, Victoria Ara-Loriente, Laura Romo-Calvo

**Affiliations:** 1 Aragón Health Service, Primary Care, Zaragoza, Spain; 2 Research Network on Preventive Activities and Health Promotion, (REDIAPP-G06-018) Nodo de Aragón, Spain; 3 Aragón Health Sciences Institute, Zaragoza, Spain; 4 Aragón Health Service, Zaragoza, Spain; 5 Hospital Provincial de Toledo, Toledo, Spain

## Abstract

**Background:**

Rotator cuff tendinopathy and subacromial impingement syndrome present complex patomechanical situations, frequent difficulties in clinical diagnosis and lack of effectiveness in treatment. Based on clinical experience, we have therefore considered the existence of another pathological entity as the possible origin of pain and dysfunction. The hypothesis of this study is to relate subacromial impingement syndrome (SIS) with myofascial pain syndrome (MPS), since myofascial trigger points (MTrPs) cause pain, functional limitation, lack of coordination and alterations in quality of movement, even prior to a tendinopathy. MTrPs can coexist with any degenerative subacromial condition. If they are not taken into consideration, they could perpetuate and aggravate the problem, hindering diagnosis and making the applied treatments ineffective.

The aims and methods of this study are related with providing evidence of the relationship that may exist between this condition and MPS in the diagnosis and treatment of rotator cuff tendonitis and/or SIS.

**Method/design:**

A descriptive transversal study will be made to find the correlation between the diagnosis of SIS and rotator cuff tendonitis, positive provocation test responses, the existence of active MTrPs and the results obtained with ultrasonography (US) and Magnetic Renonance Imaging (MRI). A randomized double blinded clinical trial will be carried out in experimental conditions: A Protocolized treatment based on active and passive joint repositioning, stabilization exercises, stretching of the periarticular shoulder muscles and postural reeducation. B. The previously described protocolized treatment, with the addition of dry needling applied to active MTrPs with the purpose of isolating the efficacy of dry needling in treatment.

**Discussion:**

This study aims to provide a new vision of shoulder pain, from the perspective of MPS. This syndrome can, by itself, account for shoulder pain and dysfunction, although it can coexist with real conditions involving the tendons.

**Trail Registration:**

ISRCTN Number: 30907460

## Background

Shoulder pain is an important cause of morbidity and has a high prevalence. It is the third most common musculoskeletal problem [1] seen in primary care consultations. Alteration of the soft or periarticular tissue is the most common cause [2-4], while bone disease, joint impairment (inflammatory or not) and joint instability [3,4] occurring to a lesser degree.

The origin of soft tissue lesions is quite varied. It can include a series of causes from subacromial or subacromiodeltoid bursitis to partial or total tearing of the rotator cuff tendon, calcific tendonitis of the rotator cuff, and tendonitis and rupture of the long biceps tendon [4-6]. Among these, the most common is found to be alterations in the rotator cuff, with SIS as the triggering physiopathological mechanism [7,8]. This is a problem regularly attended to in primary care [1,9].

Diagnosis is traditionally based on detailed history and clinical examination findings [1,4,10]. There are no conclusive tests included in the physical examination [3,11,12], the general practitioner's basic diagnostic tool, that allows obtaining an accurate diagnosis of alterations to the rotator cuf. A number of studies suggest that a diagnostic imaging test should be associated with the physical exploration [13,14]. US is a non-invasive method with a diagnostic precision [15] comparable to that of MRI for both, subacromial bursitis [13] and the different degrees of rotator cuff alteration [14,16]. Given the lack of clarity in clinical diagnosis, the existence of lesions in asymptomatic shoulders [17,18] and the scarce effectiveness of treatments [19,20], some authors consider that there may be another structure causing pain and dysfunction [21-23].

One solid hypothesis consists of relating rotator cuff tendonitis, as the most common cause of shoulder pain [1], with myofascial pain syndrome [23-25].

Myofascial pain syndrome is associated with differentiated, linear band-like hardness of the muscle containing hyperalgesic zones called MTrPs, which cause nociceptive agents such as substance P, potassium and histamine, among many others, to be released. These agents sensitize peripheral nociceptors and nociceptive neurones in the spinal dorsal horn [26]. The MTrP can be defined as a hyperirritable nodule that elicits local and, frequently, referred pain when pressured, situated in a palpable taut band formed by skeletal muscle fibres. It can be found either in the middle part of the muscle fibres (central MTrP) or in the areas where the muscle fibres attach to the tendon (attachment MTrP) [22,26]. With regard to clinical activity, MTrPs can be active or latent. Both cause dysfunction, but only active MTrPs produce spontaneous referred pain [22].

Specific mechanical stimulation of the MTrP, either by snapping palpation of by needling, can elicit a transient and isolated contraction of the fibres of the taut band called local twitch response (LTR) [27]. Despite its specificity, for a number of reasons, such us its low interrater reliability or the difficulties to elicit it in deep muscles, LTR is not considered an essential criterion for diagnosing MTrPs, but just a confirmatory observation [27].

According to Simon's integrated hypothesis [23,28], excessive release of acetylcholine in some dysfunctional motor endplates causes a contracture responsible for the appearance of the taut band in the affected muscle. The capillaries compression caused by the sarcomere shortening reduces local blood circulation, producing ischemia. The availability of oxygen and adenosine triphosphate (ATP) decreases, causing a local energy crisis. The resulting tissue suffering induces the release of sensitizing, substances that activate nociceptors cause pain and contribute to maintaining and further aggravating the dysfunction of the motor endplate [28]. All this would explain the palpatory findings and the increase of local pain sensitivity, that are commonly used as diagnostic criteria for MTrPs [23,29].

The dysfunction produced by trigger points is characterized by muscle shortening, muscle weakness local or referred inhibition, difficulty in relaxation, lack of coordination, fatigability and delayed recovery, spontaneous pain, pain on vigorous stretching and contracting [30]. Diagnosis of MTrPs is based on physical examination. There are no routine laboratory or imaging tests that objectively substantiate their existence [23], although in recent years specific needle electromyographic examination has become a way to objectively identify MTrPs [31-33]. Even more recently, magnetic resonance elastography [34] or Doppler US associated with US elastography [35] have shown potential to visualize the taut band or the MTrP.

There are currently different treatments for MTrPs [22,36]. The use of the mechanical stimulus of a needle to inactivate and/or eliminate the MTrP is increasing among physical therapists and other health practitioners [28,37] Since no substance is injected, the technique is commonly referred to as dry needling, and it has proven to be as effective as injection in the treatment of MTrPs by mechanically disrupting the MTrP and by activating pain control mechanisms at the spinal dorsal horn level [38].

We can see that diagnosis for SIS, rotator cuff tendonitis and MTrPs are all based mainly on medical history and physical examination [4,10,28].

Some provocation tests to provide evidence of tendonitis are not specific, since they consist of inducing a selective contraction of the muscle being tested and waiting for the pain response [10], which might also give positive results in case that muscle harboured a central MTrP, for pain (either local or referred) on contraction is also a common clinical feature of MTrPs [22,30].

In fact, we can find MTrPs that are prior to tendonitis [39,40]; they can coexist with all degenerative tendinopathy situations.

In short, we are faced with a pathological entity where suffering and degeneration are observed in the group of rotator cuff tendons caused by the impingement phenomenon, in which physiological movement is lost from lowering the humeral head [8] and the correct movement of the scapula [22]. By considering neuromuscular control as a key part of active movement, we are attempting to justify how a number of muscles affected by MTrPs can alter quality of movement, even before tendonitis occurs. MTrPs can coexist with all degenerative subacromial conditions, and perpetuate and aggravate the problem. They hinder diagnosis and render the given treatments ineffective.

### AIMS

The aims and design of the study cover two aspects: diagnosis and treatment of SIS and rotator cuff tendonitis.

### AIMS Regarding Diagnosis

- Identification of any correlation between the existence of active MTrPs and SIS and rotator cuff tendonitis.

- Identification of any correlation between the results of provocation tests for SIS and the existence of active MTrPs.

- Evaluation of the diagnostic reliability of provocation tests for SIS, using MRI and US as standard tests.

- Evaluation of the diagnostic reliability of physical exploration for active MTrPs, using US as a standard evidence.

### AIMS Regarding Treatment

- Evaluation of the efficacy of dry needling of active MTrPs in the treatment of SIS and rotator cuff tendonitis compared to habitual clinical protocols.

## Design

**Figure 1:**
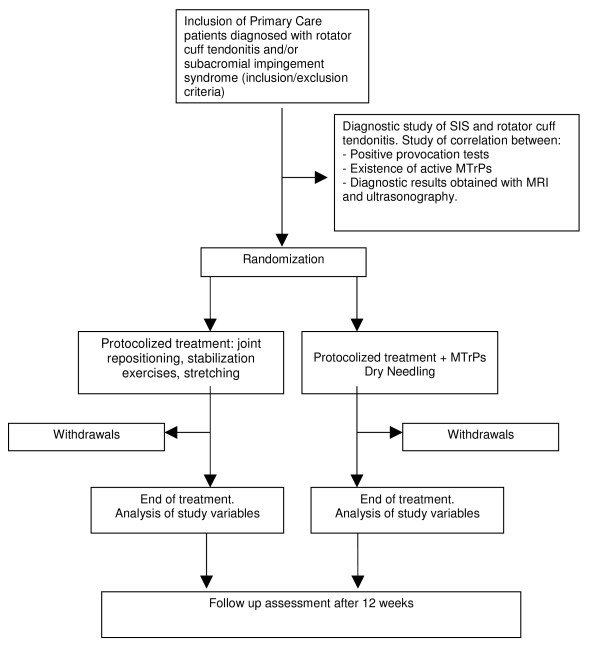
**Recruitment and project outline**.

The project outline is as follows (figure 1):

### In Relation to Diagnosis

Design: Transversal descriptive study

Study subjects: Patients belonging to 5 health centres in the city of Zaragoza (Spain) over the age of 18 who are diagnosed with rotator cuff tendonitis and/or SIS by a general practitioner (GP) and who give their consent after being informed about participation in the study. The minimum number of patients to be recruited is 132.

Inclusion criteria:

- Diagnosis of rotator cuff tendonitis and/or SIS by GP.

- Functional limitation and pain above 50% of the range of movement in flexion, abduction, and elevation in the scapular plane.

Exclusion criteria:

- Previous surgery for SIS

- Great limitation, pain or sudden loss of strength after suffering a traumatism that suggests another serious condition.

- Other red flag signs indicating systemic disease.

- Inability to attend intervention sessions or refusal to participate.

Study variables related to diagnosis are shown on the table 1:

**Table 1:** Study Variables Related to Diagnosis

General variables	Age
	Sex
	Occupation
Pain history	Period of evolution
	Background

Previous treatments	Drug type
	Period administered
	Evolution with medication

Elevation in the scapular plane	Score on Visual Analogical Scale (VAS) Location of pain

Joint restriction (digital inclinometer)	Flexion-extension movements
	Abduction-adduction movements
	Lateral-medial rotation movements

Constant Test	

Provocation tests	Neer manoeuvre
	Jobe's test
	Hawkins test
	Yocum test
	Patte's test
	Gerber test
	Drop arm test. Painful arc test

MTrP existence by means of:	Physical examination
	Ultrasonography

The following confusion variables will be controlled: osteophytes on the lower face of the acromion, existence of latent trigger points on serratus anterior, biceps braquialis and both pectoralis, sports activity and occupation involving lifting of upper limbs above 90° of flexion or abduction.

Data analysis: a descriptive analysis will be made of the defined variables followed by a bivariate analysis by means of Pearson's r, Spearman's rho or Kendall's tau-b correlations. Multivariate analysis will be performed using logistic regression, including the variables that had a significant result in the bivariate analysis. A correlation will be established between diagnosis of SIS and rotator cuff tendonitis, positive provocation tests, existence of active MTrPs and the diagnostic results obtained with US.

### In Relation to Treatment

#### Design: Random clinical trial

The subjects included in the study will be randomized (with a hidden sequence) to two sets of experimental conditions:

1. – Protocolized treatment based on active and passive joint repositioning, stabilization exercises, stretching of the periarticular shoulder muscles and postural reeducation.

2. – The previously described protocolized treatment with the addition of dry needling of active MTrPs on the supraspinatus, infraspinatus, subscapularis and teres minor muscles using Hong's fast-in and fast-out technique, once for each active MTrP and accompanied by a cold spray to reduce the painful post-needling sensation. Only one needling will be performed in each session with an interval of 8 days between sessions.

The purpose of these treatment methods is to isolate the efficacy of dry needling in the treatment of rotator cuff tendonitis and/or SIS.

Training and standardization sessions will be held for both protocols so that the intervention will be the same for all subjects in the study.

The treatment will consist of 10 physiotherapy sessions and the measuring of dependent variables will be made during the initial visit and at the end of the treatment. The final and follow-up measuring will be performed by a blind evaluator.

The sample size is 132 patients (with and error of 0.05 and a power of 80%, if a correlation of 0.25 of the sample size is taken to be 120 patients; expecting a 10% attrition, the necessary sample size would be 132 patients). The patients will be randomized to include 66 subjects in each therapy method.

The study variables related to treatment are shown on the table 2:

**Table 2:** Study Variables Related to Treatment

INDEPENDENT VARIABLE	DEPENDENT VARIABLE	
THERAPY METHOD	Joint restriction by	Flexion-extension movements
		
	digital inclinometer	Abduction-adduction movements
		
		Lateral-medial rotation movements
	
A. Protocolized treatment	Pain on elevation in the scapular plane	VAS scoring
		
		Location of pain
	
B Protocolized treatment + dry needling of MTrPs	Function Constant Test	
	
	Existence of active MTrPs by algometry	Supraspinatus
		
		Infraspinatus
		
		Subescapularis
		
		Teres minor

## Data analysis

- Multiple variance analysis techniques to evaluate the differences between pre- and post-treatment values in dependent variables for both groups. The variables with significant basal moment differences between both groups (despite randomization) that may constitute a factor of confusion will be controlled.

- Multivariate analysis to determine the influence of the treatment programme on the evolution of symptoms. Routinely used techniques in clinical trials will be employed, such as intention to treat (to manage withdrawals from either group) and survival curves, among others.

### Ethical Aspects of the Study

The study follows the standards of the Helsinki Declaration on good clinical practices and will be carried out with the approval of the Ethics Committee of Aragón.

- Patients will be required to give informed consent as a condition to participate in the study. After the completion of the study, GPs will be informed of their patients' diagnoses, existing active MTrPs and their evolution.

- Researchers taking part in the study will be required to sign a Confidentiality Agreement in which they agree to follow the procedures established in the project.

- Any information allowing patients to be identified will be encrypted for storage.

## Discussion

This study aims to provide a new vision of shoulder pain, from the perspective of MPS, a syndrome that can account for shoulder pain and dysfunction by itself, but which is perfectly compatible with the coexistence of real conditions affecting the tendons.

As previously mentioned, shoulder pain is an important cause of morbidity with a high prevalence, and a diverse and confusing background from a diagnostic perspective. GPs lack sufficient tools for its diagnosis, since it is based on a clinical examination, without conclusive tests and with few complementary tests, and its treatment (low effectiveness of the treatments described in the literature). This study aims to confirm the information obtained from the very limited literature found on the specificity and sensitivity of the provocation tests the examinater has available. Complex diagnosis complicates treatment, which is fundamental if chronicity and surgery want to be avoided.

Confirmation of the existence of active MTrPs in shoulder pain by means of their visualization with a simple diagnostic technique would allow diagnosis and, consequently, treatment to be improved.

## Competing interests

The authors declare that they have no competing interests.

## Authors' contributions

SP has made contributions to conception and design of the study, coordination work, and acquisition of data. BO has participated in the conception and design of the study and performed the statistical data base. AA has collaborated with the diagnostic imaging. OM has revised the manuscript, contributed with important intellectual content and has given final approval of the version to be published. EG has carried out the acquisition of data. MaLD has carried out the acquisition of data. EL has carried out the acquisition of data. MP has carried out the acquisition of data. VA has carried out the acquisition of data and LR has carried out the acquisition of data. All authors read and approved the final manuscript.

## Pre-publication history

The pre-publication history for this paper can be accessed here:


